# Molecular basis for the role of oncogenic histone mutations in modulating H3K36 methylation

**DOI:** 10.1038/srep43906

**Published:** 2017-03-03

**Authors:** Yinglu Zhang, Chun-Min Shan, Jiyong Wang, Kehan Bao, Liang Tong, Songtao Jia

**Affiliations:** 1 Department of Biological Sciences, Columbia University, New York, NY 10027, USA.

## Abstract

Histone H3 lysine 36 methylation (H3K36me) is critical for epigenetic regulation and mutations at or near H3K36 are associated with distinct types of cancers. H3K36M dominantly inhibits H3K36me on wild-type histones, whereas H3G34R/V selectively affects H3K36me on the same histone tail. Here we report the crystal structures of SETD2 SET domain in complex with an H3K36M peptide and SAM or SAH. There are large conformational changes in the substrate binding regions of the SET domain, and the K36M residue interacts with the catalytic pocket of SETD2. H3G34 is surrounded by a very narrow tunnel, which excludes larger amino acid side chains. H3P38 is in the *trans* configuration, and the *cis* configuration is incompatible with SETD2 binding. Finally, mutations of H3G34 or H3P38 alleviate the inhibitory effects of H3K36M on H3K36me, demonstrating that the stable interaction of H3K36M with SETD2 is critical for its inhibitory effects.

Residue-specific posttranslational methylation of histone lysines plays essential roles in every aspect of DNA metabolism such as transcription, DNA replication, recombination, and DNA damage repair^1^. Among them, methylation of H3K36 regulates diverse cellular processes and misregulation of H3K36 methyltransferases is associated with a number of human diseases^2^. Interestingly, several of the recently identified oncogenic histone mutations are at or near H3K36. For example, the majority of chondroblastomas contain an H3K36M mutation, non-brainstem gliomas frequently contain H3G34R/V mutations, and giant cell tumors of the bone recurrently contain H3G34W/L mutations^3^,^4^,^5^,^6^,^7^. Introducing an H3K36M transgene dominantly blocks H3K36me on wild type histones, whereas H3G34V or H3G34R transgenes selectively affect H3K36me on the mutated histones only^8^,^9^,^10^,^11^,^12^. In addition to these pathological mutations, alterations that prevent the isomerization of proline 38 also affect H3K36me^13^. However, the mechanism by which these mutations regulate H3K36me is not well understood.

In yeasts, a single H3K36 methyltransferase Set2 is responsible for all three degrees of H3K36 methylation^14^,^15^. In contrast, multiple H3K36 methyltransferases are present in mammals, including NSD1, NSD2/MMSET, NSD3, ASH1L, SETD2, and others^2^. NSD1, NSD2/MMSET, NSD3, and ASH1L are responsible for H3K36me2 and H3K36me1, whereas SETD2 is responsible for H3K36me3^2^,^16^,^17^,^18^,^19^. Among these H3K36 methyltransferases, SETD2 is highly conserved across species. In addition, the enzymatic activity of SETD2 is inhibited by the H3K36M mutation, and SETD2 failed to methylate histone tails containing H3G34 mutations^10^,^11^,^12^, making it ideal to further study the molecular mechanisms of oncogenic histone mutations in regulating H3K36 methylation.

Mutations of other methylated lysines into methionines also dominantly inhibit the methylation of corresponding lysine residues on wild type histones, suggesting that histone lysine-to-methionine mutations function through a similar mechanism^9^,^10^,^20^,^21^. We have previously shown that in fission yeast H3K9M-containing nucleosomes traps the H3K9 methyltransferase Clr4 at heterochromatin nucleation centers to block its spreading, therefore preventing the formation of large heterochromatin domains^20^. Similarly, immunoprecipitation of H3K27M-containing nucleosomes copurified higher amounts of the H3K27 methyltransferase complex PRC2^8^,^22^, and immunoprecipitation of H3K36M-containing nucleosomes showed higher levels of H3K36 methyltransferases such as SETD2 and NSD1^11^,^12^. However, NSD1 was not inhibited by H3K36M *in vitro*^11^, raising the question whether H3K36M also functions through sequestration of H3K36 methyltransferases to affect H3K36me.

We and others have shown *in vitro* that H3K9 methyltransferases such as Clr4 and mammalian G9a interact with K9M-containing histone H3 peptides in the presence of S-adenosylmethionine (SAM), the methyl donor for histone methyltransferases^20^,^23^. Similarly, the interaction between the H3K27 methyltransferase PRC2 and an H3K27M peptide is also dependent on SAM^24^. Such interactions allow the crystallization of the ternary complexes for structural analyses^20^,^23^,^24^. In this study, we solved the structures of an H3K36M peptide in complex with the catalytic SET domain of mammalian SETD2 and S-adenosylmethionine (SAM), or S-adenosyl-L-homocysteine (SAH), and revealed the detailed molecular mechanism by which SETD2 interacts with the histone H3 tail. The nearly identical SAM and SAH containing structures suggest that the methyl group of SAM is not important for interactions between SETD2 and H3K36M, which is different from other lysine-to-methionine mutations. Using the structure as a guide, we were able to generate mutations that reduce interactions between H3K36M and its methyltransferases, and show that the sequestration of SETD2/Set2 by H3K36M-contianing nucleosomes is essential for the inhibitory effects of the H3K36M mutation.

## Results

### The structure of the SETD2-H3K36M-SAM complex

**Figure 1:**
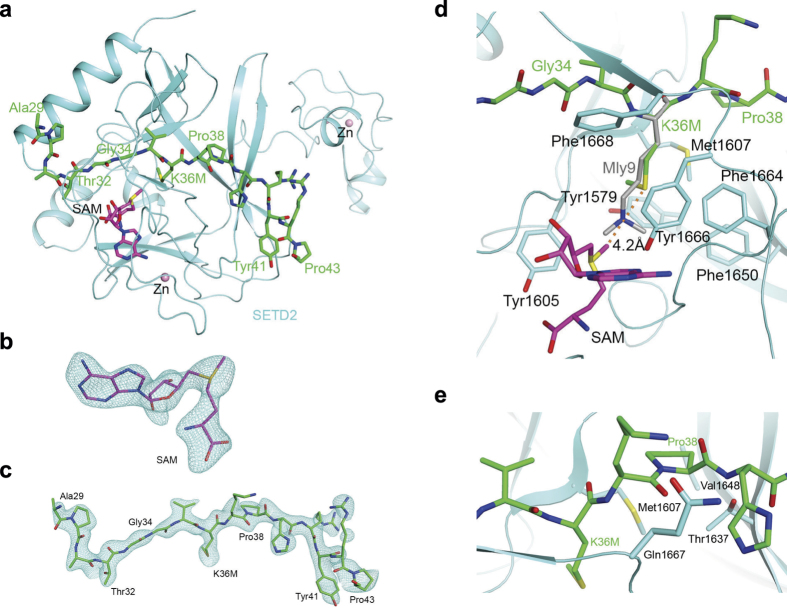
The structure of the SET domain of SETD2 in complex with an H3K36M peptide and SAM. (**a**) The overall structure of the SET domain in complex with the H3K36M peptide. The SET domain is shown in cyan, the H3K36M peptide in green stick models and SAM in magenta. Several zinc ions bound to the protein are shown as pink spheres. (**b**) Omit F_o_−F_c_ electron density at 2.42 Å resolution for SAM, contoured at 3σ. (**c**) Omit F_o_−F_c_ electron density at 2.42 Å resolution for the H3K36M peptide, contoured at 3σ. (**d**) Detailed interactions between the SETD2 SET domain (cyan) and the H3K36M peptide (green) near K36M. The distance between the methyl group of SAM and the sulfur atom of K36M is indicated by the dashed line. The bound position of dimethylated H3K9 (labeled Mly9) peptide to GLP1^25^ is also shown (gray). (**e**) Pro38 assumes a *trans* configuration and has favorable interactions with the SET domain. All structure figures were produced with PyMOL (www.pymol.org).

It has been shown that H3K36M-containing nucleosomes or peptides pull down SETD2 from nuclear extracts more efficiently than their wild type counterpart^11^,^12^. To understand the molecular details of the interactions, we generated recombinant SET domain of SETD2 (residue 1435–1711, encompassing the AWS, SET, and post-SET regions) and an H3K36M peptide (29-APATGGVMKPHRYRP-43). We then determined the 2.42 Å resolution crystal structure of the SET domain of SETD2 in complex with the H3K36M peptide and SAM (Fig. 1a). The atomic model has good agreement with the X-ray diffraction data and the expected bond lengths, bond angles and other geometric parameters (Table 1). All the residues are located in the preferred regions of the Ramachandran plot (data not shown).

Clear electron density was observed for the entire H3K36M peptide and SAM (Fig. 1b and c) based on the crystallographic analysis. Residues 32–43 of the peptide assume an extended conformation, and residues 33–35 have anti-parallel interaction with a strand (residues 1668–1670) in the post-SET motif of SETD2. The last two residues of the peptide are projected away from the rest of SETD2. The conformation of Pro43 is likely stabilized through van der Waals interactions with the side chain of Tyr41 (Fig. 1a), and the Arg42 side chain is stabilized by crystal packing.

### The interaction of H3K36M with SETD2

The K36M residue of histone H3 is located in the active site of SETD2, confirmed by the fact that a doubly methylated lysine residue (Mly9) of the H3K9 peptide is bound at a similar position in the SET domain of its methyltransferase GLP-1 (Fig. 1d)^25^. The methionine side chain does not extend as deeply into the pocket as compared to the lysine side chain. It is surrounded by a collection of aromatic side chains (Tyr1579, Tyr1605, Phe1650, Phe1664, Tyr1666, Phe1668) as well as Met1607 from SETD2. The methyl group of K36M is pointed toward the side chain of Tyr1579, away from the direction of the Lys substrate. The binding mode for methionine is similar to that of the recently solved structures of G9a-K9M and PRC2-K27M^20^,^23^,^24^, where the same residues that interact with the substrate lysine also interact with the mutated methionine residue.

The methyl group of the cofactor SAM is pointed toward the K36M side chain of the peptide, but at a distance of 4.2 Å to its sulfur atom and 4.3 Å to its methyl group (Fig. 1d), suggesting it does not directly mediate interaction with the methionine side chain. Therefore, the contribution of SAM to the interaction between SETD2 and H3K36M is mainly to stabilize the proper folding of the SET domain. Our structural data indicated that the electron density for the methyl group is weaker compared to the rest of SAM (Fig. 1c), suggesting that a mixture of SAM and SAH are present in this structure. As we only added SAM during crystallization, SAH is likely a degradation product, as has been observed before^26^. We obtained another ternary complex structure at 2.4 Å resolution (Table 1) where the electron density for the cofactor is more consistent with SAH (Extended data, Fig. 1). While our manuscript was under review, a similar structure of the SETD2-H3K36M-SAH complex was published^27^. The SAM and SAH containing ternary complex structures are nearly identical (Extended data, Fig. 1). Therefore, the contribution of SAM and SAH to the interaction between SETD2 and H3K36M could be highly similar, which possibly stabilize the proper folding of the SET domain.

### Large conformational changes within SETD2 upon peptide binding

**Figure 2:**
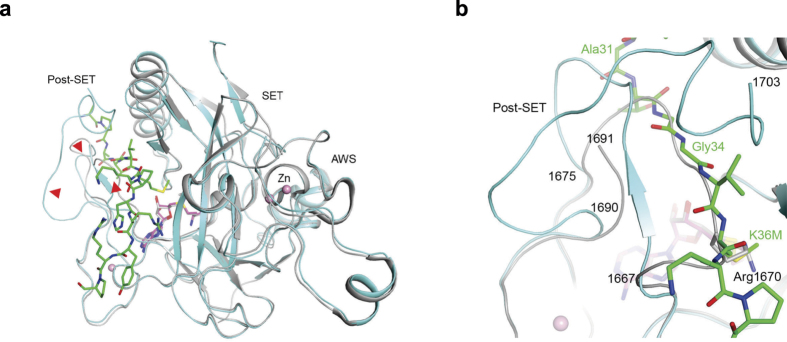
Extensive conformational changes in SETD2 SET domain upon H3K36M peptide and SAM binding. (**a**) Overlay of the structure of SETD2 SET domain (cyan) in complex with H3K36M peptide (green) and SAM (magenta) with that of the SET domain in complex with SAH (gray)^26^. Red arrowheads point to regions of large conformational differences between the two structures, in the post-SET motif. (**b**) The binding site for the substrate peptide (green) is occupied by a loop of the protein in the free SET domain structure (gray)^26^, due to large conformational changes for residues 1667–1675 and those at the C-terminus of the SET domain.

Compared to the structure of SETD2 in a binary complex with SAH^26^,^27^, the overall structures of the AWS motif and the SET domain are similar (Fig. 2a). But there are significant conformational changes for the post-SET motif of SETD2 upon binding of the peptide, with residues 1667–1675 assuming different conformations in the two structures (Fig. 2a). Especially, residues 1669–1673 in the SAH binary complex directly overlap with residues 33–36 of the H3K36M peptide, and the side chain of Arg1670 is situated in the binding site for K36M (Fig. 2b)^26^. The conformation of residues 1667–1675 in the structure of SETD2 in complex with the inhibitor N-propyl sinefungin (Pr-SNF)^26^ is more similar to that observed here (because the propyl group would clash with Arg1670 of the SAH binary complex), but residues 1669–1673 would still clash with the H3K36M peptide (Extended data Fig. 2). In addition, residues at the C-terminus (1692–1702) are disordered in the SAH binary complex and residues 1690–1691 have different conformations.

### The interaction of H3G34 with SETD2

**Figure 3:**
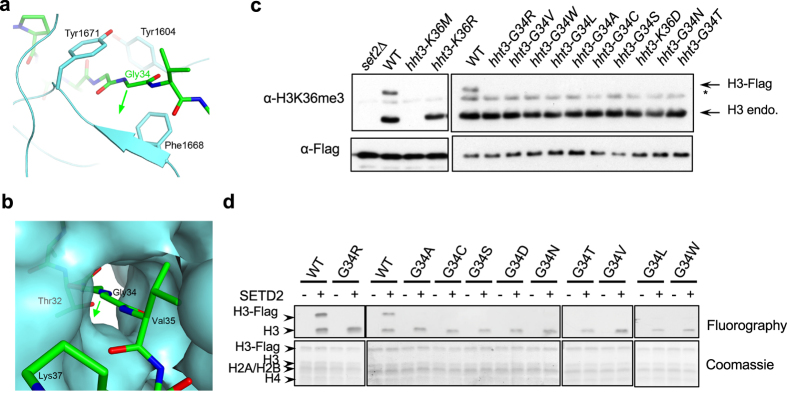
Gly34 is located in a tight tunnel that excludes large side chains. (**a**) Detailed interactions between Gly34 and the SET domain. The green arrow points to the direction of any side chain at this residue, which would clash with residues 1668–1669 of the SET domain. (**b**) Molecular surface of the SETD2 SET domain near Gly34, showing that the residue is located in a tight tunnel and is completely shielded from the solvent. (**c**) Western blot analyses of H3K36me3 and Flag levels. The mutant histone H3s contain a Flag tag, resulting in slower mobility. (**d**) *In vitro* histone methyltransferase assay with recombinant SETD2 and heterotypic nucleosomes containing H3G34 mutations.

Besides allowing the peptide to bind, another important consequence of these structural changes is that residues Gly33-Gly34 of the peptide are completely buried in the complex. Most importantly, Gly34 of the peptide is surrounded by a very narrow tunnel, flanked by Phe1668 on one side and Tyr1671 on the other (Fig. 3a and b) and mutations of these two residues into alanine completely abolished the enzymatic activity of SETD2 *in vitro*^26^. There is essentially no space to accommodate larger side chains at Gly34, such as those of oncogenic histone mutations at H3G34 (Val, Leu, Arg, and Trp).

To further test this hypothesis, we examined the effects of mutating H3G34 *in vivo*. Since there are multiple histone H3K36 methyltransferases in mammals, which might not recognize H3K36 in the same fashion, we performed mutational analyses in fission yeast, which contains only a single H3K36 methyltransferase Set2^14^. Set2 is highly conserved with human SETD2, and key residues required for the interactions with H3G34 and H3K36M are also conserved (Extended data, Fig. 3). This organism contains three histone H3 genes, *hht1*^+^, *hht2*^+^, and *hht3*^+^, with identical protein sequences^28^. We generated a Flag-tagged version of H3 at the endogenous *hht3*^+^ chromosomal locus (*hht3-Flag*), which resulted in a higher molecular weight form of H3 that can be distinguished from endogenous H3. These cells still contain two wild type copies of histone H3 genes (*hht1*^+^ and *hht2*^+^). Western blot analyses showed that both forms of H3 contain H3K36me3, which were absent in *set2∆* (Fig. 3c). Moreover, *hht3-K36M* dominantly blocked H3K36me3 at endogenous H3, whereas *hht3-K36R* only affected H3K36me3 on the mutant H3 (Fig. 3c), consistent with observations in mammalian systems^8^,^9^,^10^,^11^,^12^. We then introduced H3G34 mutations at the endogenous *hht3* locus, including the oncogenic Arg, Val, Leu, and Trp mutations or mutations to other amino acids with one or two carbon side chains (Ala, Cys, Ser, Asp, Asn, and Thr). All of these mutations severely affected H3K36me3 on the mutated histone H3 *in cis*, but had little effects on H3K36me3 on endogenous H3 (Fig. 3c).

Given that H3G34 is very close to H3K36, it is possible that the loss of H3K36me3 on the mutated histones is due to effects on antibody recognition. To rule out this possibility, we performed *in vitro* histone methyltransferase assays with recombinant SET domain of SETD2 and affinity purified heterotypic nucleosomes from fission yeast strains that contain both Flag-tagged mutant H3 and wild type endogenous H3. We then examined the incorporation of radioactively labeled methyl group by fluorography. The Flag-tagged wild type H3 is methylated as efficiently as the endogenous H3 by SETD2 (Fig. 3d). In contrast, Flag-tagged H3 containing mutations at H3G34 all showed severely reduced methylation (Fig. 3d). Therefore, the G34 position has little tolerance for mutations, which might explain why H3G34 is a hotspot for oncogenic histone mutations.

**Figure 4:**
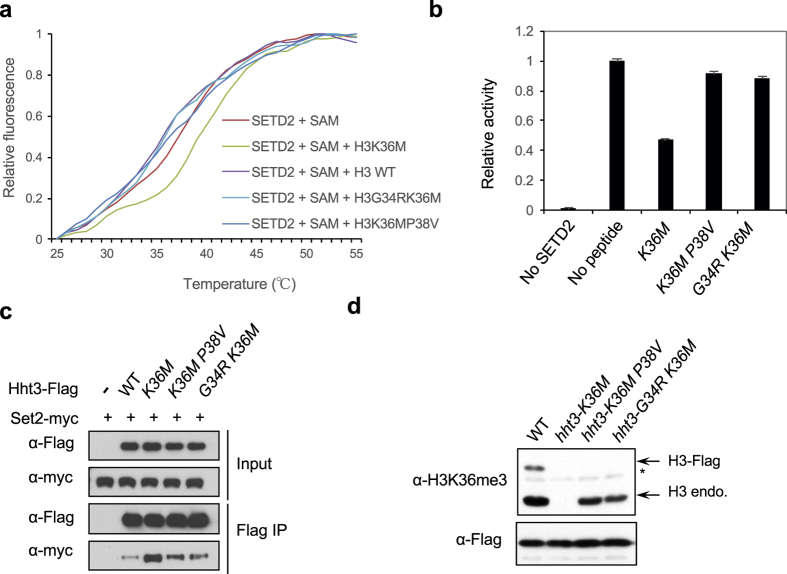
The effects of H3K36M mutation can be modulated by mutations in H3G34 or H3P38. (**a**) Thermal shift assay with recombinant SET domain of SETD2 and 1 mM of histone peptides in the presence of 0.4 mM of SAM. (**b**) *In vitro* histone methyltransferase assay with recombinant SET domain of SETD2 and histone H3 tail peptides. The incorporation of a radioactively labeled methyl group was quantified by scintillation counting. The activity without peptide was set to one. Error bars represent standard deviation of two replicates. (**c**) Co-immunoprecipitation analysis to examine the interaction between Set2-myc and histones containing different mutations. Only one of the three copies of histones was Flag-tagged and contains mutations. (**d**) Western blot analyses of H3K36me3 and Flag levels. Only one of the three copies of histones was Flag-tagged and contains mutations.

We also tested the requirement of residues restricting the G34 recognition tunnel by mutating Tyr301 of Set2, which correspond to Tyr1671 of SETD2. Consistent with our hypothesis, the Y301L mutation, which has an intermediate side chain, mildly affected H3K36me3. In contrast, a Y301A mutation, which has a short side chain, reduced H3K36me3 levels even further (Extended data, Fig. 4), suggesting that a narrow G34 access channel is critical for recognition of the H3 tail by Set2.

### The interaction of H3P38 with SETD2

Isomerization of H3P38 by proline isomerase Frp4, which converts proline between *trans* and *cis* configurations, regulates H3K36 methylation by Set2^13^. As seen in our structure, Pro38 of H3 is bound in the *trans* configuration (Fig. 1e), causing a ~90° twist in the backbone of the peptide. This allows residues C-terminal to P38 to make extensive contacts with SETD2. In contrast, a *cis* configuration of Pro38 is incompatible with the binding of the peptide to SETD2, as it will cause severe steric clashes between the histone tail and SETD2. Therefore, Pro38 isomerization directly contributes to the stable binding of histone substrate by SETD2.

### Mutations in H3G34 or H3P38 affects interaction between H3K36M and its methyltransferases

We then tested whether G34 or P38 is required for the interaction between SETD2 and the histone H3 tail. Given that there is no detectable interaction between SETD2 and the wild type H3 peptide, we generated G34R or P38V mutation (which abolishes H3K36me3 *in cis* in budding yeast^13^) in the context of the H3K36M peptide. Thermal shift assay indicated that the H3G34RK36M and H3K36MP38V peptides could not stabilize SETD2 as efficiently as H3K36M in the presence SAM (Fig. 4a and Extended data Fig. 5). Therefore, the G34R or P38V mutation affects the interaction between H3K36M and SETD2.

We then tested the effects of G34R or P38V mutation on the ability of H3K36M peptide to inhibit SETD2 enzymatic activity *in vitro*. Consistent with previous findings, the H3K36M peptide inhibited the activity of SETD2 on recombinant nucleosomes (Fig. 4b). In contrast, H3K36MP38V or H3K36MG34R peptide only had minor effects on SETD2 activity *in vitro* (Fig. 4b). Therefore, the stable interaction between H3K36M and SETD2 is required for its inhibitory effects.

To further examine the effects of G34R and P38V on the ability of H3K36M to inhibit H3K36me3 *in vivo*, we introduced K36MP38V or G34RK36M mutations into the endogenous *hht3* locus in fission yeast. These mutants were efficiently incorporated into chromatin (Extended data Fig. 6). Co-immunoprecipitation analysis of cells expressing Set2-myc and Hht3-K36M-Flag showed that Set2-myc interacted strongly with H3K36M containing nucleosomes compared to wild type nucleosomes, consistent with findings in mammalian cells^11^,^12^. However, the interaction was reduced in Hht3-K36MP38V or Hht3-G34RK36M expressing cells (Fig. 4c). Moreover, ChIP analysis showed that the localization of Set2 to one of its target genes *tef3* was increased in *hht3-K36M* cells, but reduced in *hht3-G34RK36M* or *hht3-K36MP38V* cells (Extended data Fig. 7). Consistent with the idea that the sequestration of H3K36 methyltransferases is responsible for the dominant effects of H3K36M, we found that H3K36me3 levels on wild type histones were significantly restored in *hht3-G34RK36M* and *hht3-K36MP38V* cells (Fig. 4d).

## Discussion

In this study, we solved the structures of a histone H3K36 methyltransferase SETD2 in complex with a histone H3K36M peptide and SAM or SAH, which provides the molecular details about how this family of enzymes recognizes their histone substrate. The structural and functional data explain why many of the oncogenic mutations are concentrated at Gly34, and how they block H3K36 methylation. It also provides the molecular basis of Pro38 isomerization in regulating H3K36me. We further demonstrated the role of modulating H3K36M-H3K36 methyltransferase interactions in mitigating the effects of the oncogenic H3K36M mutation, which might be further explored to alleviate the toxicity of H3K36M mutation in cancer cells.

Previous studies on the interaction between H3K9M and its methyltransferases such as Clr4 and G9a demonstrate that the interactions are critically dependent on the cofactor SAM. In contrast, the product of methylation reaction, SAH, has relatively little effects^20^,^23^. Therefore, it is interesting that we and others obtained the structure of SETD2 in complex with H3K36M and SAH (Extended data, Fig. 2)^27^. In fact, our SETD2-H3K36M-SAM structure showed that the methyl group of SAM has a lower electron density, most likely due to the degradation of SAM during crystallization. Such heterogeneity has no effects on the entire complex structure, suggesting that the methyl group does not contribute to the interaction. How the methyl group enhances the interaction between G9a and H3K9M is unknown, as the structure revealed that SAM is not directly involved in interaction with the methionine side chain either^20^,^23^. It is possible that H3K9M and H3K36M function through slightly different mechanisms in inhibiting the enzymatic activity of their corresponding methyltransferases. Such a difference might also have functional consequences as H3K9M can only inhibit Clr4 and G9a before the methylation reaction when the methyltransferases are still bound by SAM, whereas H3K36M can sequester its methyltransferases even after they have methylated wild type histones.

H3K9 methyltransferases G9a and Clr4 are inhibited by the H3K9M mutation and the H3K27 methyltransferase PRC2 is inhibited by the H3K27M mutation^10^,^20^,^23^. These enzymes all show increased association with their corresponding lysine-to-methionine mutant histones^8^,^11^,^12^,^22^. The prevailing hypothesis is that the increased association of lysine-to-methionine mutant histones with their corresponding histone methyltransferases sequesters the enzymes^12^,^20^,^23^,^24^. Indeed, we previously showed that reducing the interaction between H3K9M and Clr4 was able to alleviate the inhibitory effects of H3K9M^20^. Interestingly, some H3K36 histone methyltransferases, such as NSD1 and ASHL1, are not inhibited by the H3K36M mutation^11^, but nonetheless show increased association with H3K36M containing nucleosomes^11^,^12^, therefore raising the possibility that mechanisms other than sequestration are responsible for the inhibitory effects of H3K36M. Our structural analyses revealed the essential roles of H3G34 and H3P38 in mediating the interaction of the H3 tail, thus providing an opportunity to test the contribution of increased association with H3K36 methyltransferase for the inhibitory effects of H3K36M. Indeed, additional mutations of H3G34 and H3P38V reduced the interaction between H3K36M and H3K36 methyltransferases and alleviate the effects of H3K36M both *in vitro* and *in vivo*. Therefore, consistent with other lysine-to-methionine mutations H3K36M also functions through trapping of SETD2/Set2.

It is not clear why SETD2 and NSD2 are inhibited by H3K36M, but NSD1 and ASHL1 are not. Comparison of H3K36 methyltransferases shows that residues mediating interactions with H3K36M are highly conserved (Extended Data, Fig. 4). The structure of NSD1 in complex with SAM showed that these residues also form a hydrophobic cage nearly identical to that of SETD2^17^. Even more striking is that these residues are identical between NSD1 and NSD2, which are differentially inhibited by H3K36M^11^. Given that NSD1 also preferentially associates with H3K36M containing nucleosomes^12^, it is likely that NSD1 escapes the inhibitory effects of H3K36M through a mechanism different from regulating enzyme sequestration. It will be informative to obtain the structure of NSD1 in complex with H3K36M to determine the detailed interaction differences from SETD2. Such information will be important for understanding the mechanism by which the H3K36M mutation affects H3K36me and might offer new opportunities to alleviate the effects of H3K36M in cancer cells.

## Methods

### Protein expression and purification

Human SETD2 (residue 1435–1711) was sub-cloned into the pET28-MHL vector. The recombinant protein, with an N-terminal hexa-histidine tag, was over-expressed in *E. coli* BL21 (DE3) Star cells (Novagen), which were induced with 1 mM IPTG and allowed to grow at 15 °C for 16–20 h. The soluble protein was purified by nickel-charged immobilized-metal affinity chromatography, ion-exchange chromatography and gel filtration chromatography. The purified protein was concentrated and stored at −80 °C in a buffer containing 20 mM PIPES (pH 6.5), 250 mM NaCl, and 10 mM DTT. The His-tag was not removed for crystallization.

### Protein crystallization

Crystals of SETD2-SAM-H3K36M and SETD2-SAH-H3K36M complexes were grown at 20 °C with the sitting-drop vapor diffusion method. SETD2 protein solution was at 10 mg/ml concentration, and the protein was incubated with SAM (Sigma) and H3K36M peptide (Eurogentec) at a molar ratio of 1:10:10. The reservoir solution contained 0.1 M KSCN and 24% (v/v) PEG 2000 MME. Fully-grown crystals were obtained one day after set-up. The crystals were cryo-protected in the reservoir solution supplemented with 10% (v/v) glycerol and flash-frozen in liquid nitrogen for data collection at 100 K.

### Data collection and processing

X-ray diffraction data set on a SETD2-SAM-H3K36M complex crystal was collected using a Saturn944HG CCD mounted on a Rigaku Micromax-003 X-ray generator. X-ray diffraction data set on a SETD2-SAH-H3K36M complex crystal was collected at the Advanced Photon Source beamline NE-CAT 24-ID-E using an ADSC Q315r detector. The diffraction images were processed and scaled with the HKL-2000 package^29^.

### Structure determination and refinement

The structures of the SETD2-SAM-H3K36M complex and the SETD2-SAH-H3K36M complex were solved by the molecular replacement method with the program Phaser-MR in PHENIX^30^. The crystal structure of human SETD2 in complex with SAH (PDB entry code 4H12) was used as the search model. The phases were used by program PHENIX^31^ for automatic model building. Manual model rebuilding was carried out with Coot^32^. The structure refinement was performed with the program PHENIX^31^, with translation, libration, and screw-rotation (TLS) parameters. The data processing and refinement statistics are summarized in Table 1.

### Thermal shift assay

The thermal stability of the SET domain of SETD2 with ligands at different concentrations was analyzed at various temperatures using the Mx3005 P Real-Time PCR system (Stratagene). Purified SETD2 protein (5 μM) was titrated with SAM (Sigma) at 0, 0.4, 1 mM concentrations, H3K36M (29–43) peptide (Anaspec) at 0, 0.4, 1 mM, and wild-type histone H3 peptide (29–43) (Biomatik) as well as its double mutant (H3G34RK36M, H3K36MP38V) (Biomatik) at 0 and 1 mM concentrations. All assays were performed in duplicate for each sample and contained final concentrations of 20 mM PIPES (pH 6.5), 400 mM NaCl and 10 mM DTT. Reactions were incubated on ice for 30 min and mixed with the fluorescence dye (SYPRO orange; Invitrogen) for monitoring protein unfolding. The temperature was increased from 25 to 99 °C in 1 °C intervals over a 75-min period. Fluorescence values for each curve were normalized to the maximum and the minimum of the curve.

### Fission yeast strain construction

Yeast strains containing epitope-tagged Set2 and histone H3 and their mutants were generated by a PCR-based module method. All other strains were constructed through genetic crosses. A list of yeast strains used is provided in Extended data Table 1.

### *In vitro* histone methyltransferase assays

Recombinant 6xHis tagged SET domain of human SETD2 (1347–1711) was purified from *E. coli* using Talon beads according to manufacturer’s protocol (Clontech). Wild type histone H3 peptide (29–43) and its mutants (H3K36M, H3K36MP38V, H3G34RK36M) with C-terminal biotin were synthesized by Biomatik at 90% purity. Histone methyltransferase assays measuring the inhibitory effects of histone peptides were performed with 0.1 μg recombinant SET domain of SETD2 and 1 μg recombinant human nucleosomes (Epicypher) in a histone methyltransferase buffer (50 mM Tris, pH 8.8, 1 mM EDTA, 0.5 mM DTT, 0.2 mM PMSF) supplemented with 2 μM [^3^H]-labeled SAM for two hours at 30 °C. The histone peptides (final concentration of 100 μM) were incubated with SETD2 for 15 minutes at 30 °C before the addition of nucleosomes as substrates. The reactions were stopped by mixing with 2xSDS loading dye and resolved by 15% SDS-PAGE. The gels are subjected to Coomassie staining to visualize proteins and then treated with EN3HANCE (Perkin Elmer) to visualize radioactively labeled substrates. For quantification, the reactions were spotted onto P81 nitrocellulose paper (Reaction Biology), washed four times with 100 mM sodium bicarbonate and one time with acetone and air-dried. Scintillation fluid was added and the amounts of radioactivity were counted using a scintillation counter.

Histone methyltransferase assays with heterotypic fission yeast nucleosomes were performed with 0.35 μg recombinant SET domain of SETD2 (1347–1711) in histone methyltransferase buffer supplemented with 0.5 μM [^3^H]-labeled SAM for one hour at 30 °C. The reactions were stopped by mixing with 2xSDS loading dye and resolved by 15% SDS-PAGE. The gels were subjected to Coomassie staining to visualize the proteins and then treated with EN3HANCE (Perkin Elmer) to visualize radioactively labeled substrates. To obtain the heterotypic nucleosomes, Hht3-Flag expressing cells were lysed with a beadbeater in lysis buffer (150 mM HEPES-KOH pH 7.6, 1 mM EDTA, 300 mM KCl). The lysates were sonicated for a total of 10 minutes to reduce chromatin size to an average of 350 base pairs. Flag-agarose beads were then added to pull down Hht3-Flag containing nucleosomes. The beads were washed four times with lysis buffer and washed with histone methyltransferase buffer before used in histone methyltransferase reactions.

### Co-immunoprecipitation analyses

Two liters of exponentially growing yeast cells were harvested, washed with 2xHC buffer (300 mM HEPES-KOH pH 7.6, 2 mM EDTA, 100 mM KCl, 20% glycerol, and 10 mM β-mercaptoethanol) and frozen in liquid nitrogen. Crude cell extracts were prepared by vigorously blending frozen yeast cells with dry ice using a household blender, followed by incubation with 10 ml 1xHC buffer containing 250 mM KCl and 1 mM PMSF for 30 min. The lysate was sonicated to fragment chromatin and then cleared by centrifugation at 20,000 rpm for one hour. The supernatants were incubated with Flag-agarose and washed four times with 1xHC containing 250 mM KCl. Bound proteins were resolved by SDS-PAGE, followed by Western blot analyses with Myc (Santa Cruz Biotechnology) and Flag (Sigma) antibodies.

### Chromatin immunoprecipitation (ChIP) analyses

Log-phase yeast cells were incubated at 18 °C for one hour and then fixed for 30 minutes in 3% freshly made formaldehyde. The cells were pelleted and washed with PBS (phosphate buffered saline) before resuspended in ChIP lysis buffer (50 mM HEPES-KOH, pH7.5, 140 mM NaCl, 1% Triton X-100, 0.1% Deoxycholate, and 1 mM PMSF). Ice cold glass beads were added and the mixtures were vigorously disrupted in a beadbeater. The lysates were collected and subjected to sonication to reduce chromatin size to 500–1000 base pairs. The cleared cell lysates were incubated with Flag-agarose beads (Sigma) overnight at 4 °C. The beads were then washed with ChIP lysis buffer twice, ChIP lysis buffer containing 0.5 M NaCl, Wash buffer (10 mM Tris, pH8.0, 250 mM LiCl, 0.5% NP-40, 0.5% Deoxycholate, 1 mM EDTA), and TE (50 mM Tris pH8.0, 1 mM EDTA). The bound chromatin fragments were eluted with TES (50 mM Tris pH8.0, 1 mM EDTA, 1% SDS) and the crosslinking was reversed by incubating at 65 °C overnight. The protein-DNA mixtures were then subjected to proteinase K treatment and phenol:chloroform extraction before the DNA was precipitated by ethanol.

Quantitative real-time PCR (qPCR) was performed with Maxima SYBR Green qPCR Master Mix (Fermentas) in a StepOne Plus Real-Time PCR System (Applied Biosystems). DNA serial dilutions were used as templates to generate a standard curve of amplification for each pair of primers, and the relative concentration of target sequence was calculated accordingly. A fragment within the heterochromatic silent mating-type locus was used as a reference to calculate the enrichment of ChIP over WCE for each target sequence. The numbers are averages of three experiments and error bars represent standard deviation. A list of DNA oligos used was provided in Extended data Table 2.

### Western blot analyses

Cell lysates were prepared by lysing yeast cells with a beadbeater in ChIP lysis buffer. The lysates were sonicated for a total of 10 minutes to fragment chromatin. The cleared lysates were mixed with 2xSDS loading dye and resolved by SDS-PAGE. Western blot analyses were performed with H3K36me3 (Abcam) and Flag (Sigma) antibodies.

### Chromatin fractionation assay

Cells were harvested and resuspended in PEMS (100 mM PIPES, 10 mM EGTA, 10 mM MgSO_4_, 1.2 M sorbitol). Zymolyase 100 T was added to a final concentration of 1 mg/ml and incubated at 37 °C for 20 minutes to digest the cell wall. The cells were washed twice with PEMS and then lysed with PBS containing 1.5% triton X-100 and 1 mM PMSF (whole cell extract). The lysates were centrifuged at 16,000 g for 15 minutes at 4 °C to obtain supernatant (soluble) and pellet (chromatin) fractions. Proteins were separated by SDS-PAGE, and Western blot analyses were performed with Tubulin or Flag antibodies.

## Additional Information

**How to cite this article**: Zhang, Y. *et al*. Molecular basis for the role of oncogenic histone mutations in modulating H3K36 methylation. *Sci. Rep.* **7**, 43906; doi: 10.1038/srep43906 (2017).

**Publisher's note:** Springer Nature remains neutral with regard to jurisdictional claims in published maps and institutional affiliations.

## Supplementary Material

Supplementary Information

## Figures and Tables

**Table 1:** Data collection and refinement statistics.

	SETD2+SAM+H3K36M	SETD2+SAH+H3K36M
**Data collection**		
Space group	*P*2_1_2_1_2_1_	*P*2_1_2_1_2_1_
Cell dimensions		
a, b, c (Å)	58.5, 76.3, 77.2	60.3, 77.2, 76.5
α, β, γ (°)	90, 90, 90	90, 90, 90
Resolution range (Å)^1^	30–2.42 (2.51–2.42)	40–2.4 (2.49–2.4)
Measured reflections	71016	77604
Unique reflections	13529	14491
*R*_merge_ (%)	10.0 (47.6)	8.5 (42.9)
I/σI	14.1 (3.2)	18.8 (3.7)
Completeness (%)	98.1 (96.9)	99.7 (99.9)
Redundancy	5.2 (5.3)	5.4 (5.3)
**Refinement**		
Resolution range (Å)	30–2.42 (2.60–2.42)	40–2.4 (2.58–2.4)
No. reflections	13077	14082
*R*_work_ (%)	20.4 (28.3)	19.5 (24.6)
*R*_free_ (%)	26.9 (35.4)	25.7 (35.2)
No. atoms		
Protein	1971	1971
Cofactor	27	26
Zn	3	3
Water	36	85
B-factors		
Protein	35.7	40.9
Cofactor	25.8	34.6
Water	29.4	37.3
RMS deviations		
Bond lengths (Å)	0.016	0.008
Bond angles (°)	1.4	0.80
Ramachandran plot residues (%)		
Most favored regions	98.03	95.28
Additional allowed regions	1.97	4.72

^1^The numbers in parentheses are for the highest resolution shell.
