# Supplementary material for: Molecular basis for the role of oncogenic histone mutations in modulating H3K36 methylation
Source: Sci Rep. 2017 Mar 3;7:43906. doi: 10.1038/srep43906 (PMC5335568; doi:10.1038/srep43906)
Supplement: Supplementary Information [file srep43906-s1.pdf]

Molecular basis for the role of oncogenic histone mutations in modulating H3K36 methylation

Yinglu Zhang[1,2], Chunmin Shan[1,2], Jiyong Wang[1,2], Kehan Bao[1], Liang Tong[1,3], and Songtao Jia[1,3]

[1]Department of Biological Sciences, Columbia University, New York, NY 10027

[2]These authors contributed equally to this work

[3]Corresponding authors: ltong@columbia.edu, jia@biology.columbia.edu

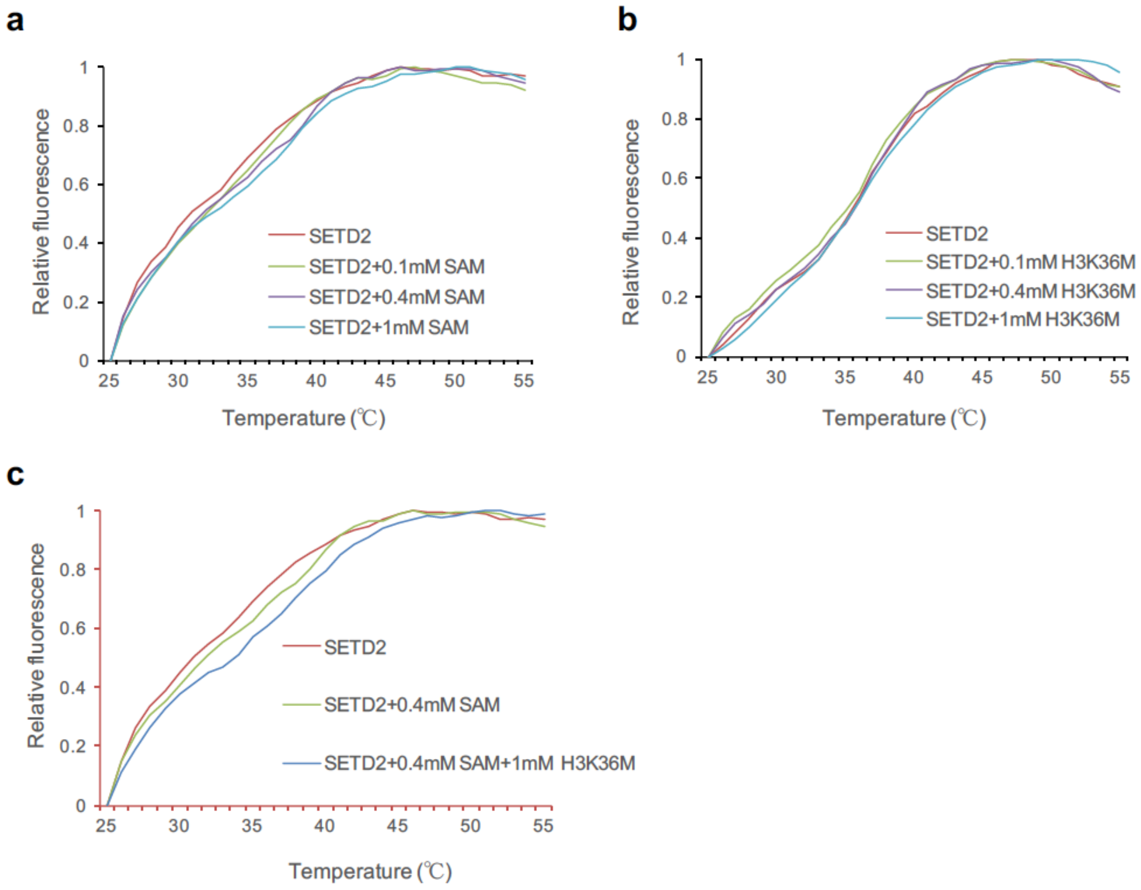

Extended data Figure 1. Thermal shift assay to measure the interaction between the SET domain of SETD2, SAM and an H3K36M peptide.

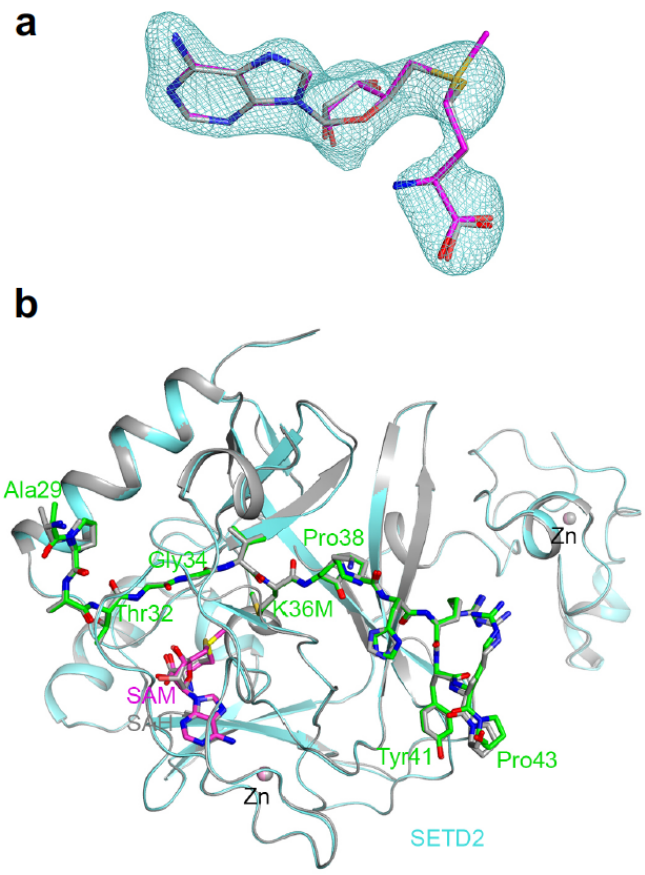

Extended data Figure 2. The structure of the SET domain of SETD2 in complex with an H3K36M peptide and SAH. (a) Omit F_o–F_c electron density at 2.4 Å resolution for SAH (in gray for carbon atoms), contoured at 3σ. The bound position of SAM is also shown (magenta) and is nearly identical. (b) The overall structure of the SET domain in complex with the H3K36M peptide and SAH (in gray) is essentially the same as that of the SAM complex (SET domain in cyan, H3K36M peptide in green stick models and SAM in magenta).

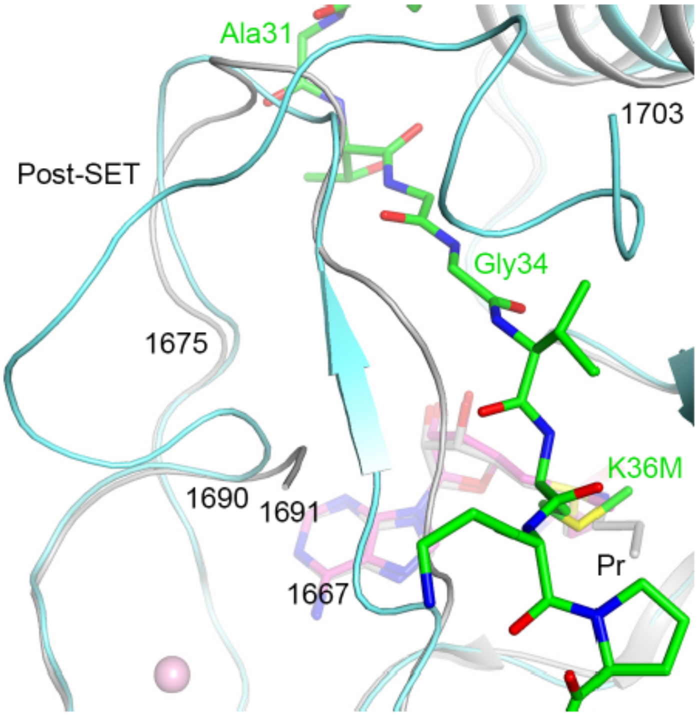

Extended data Figure 3. Overlay of the structure of SETD2 SET domain (cyan) in complex with H3K36M peptide (green) and SAM (magenta) with that of the SET domain in complex with the inhibitor N-propyl sinefungin (Pr-SNF) (gray)[25]. There are still large conformational differences, although the steric clash between the H3K36M peptide and the post-SET motif is not as severe. The propyl group of Pr-SNF is labeled Pr.

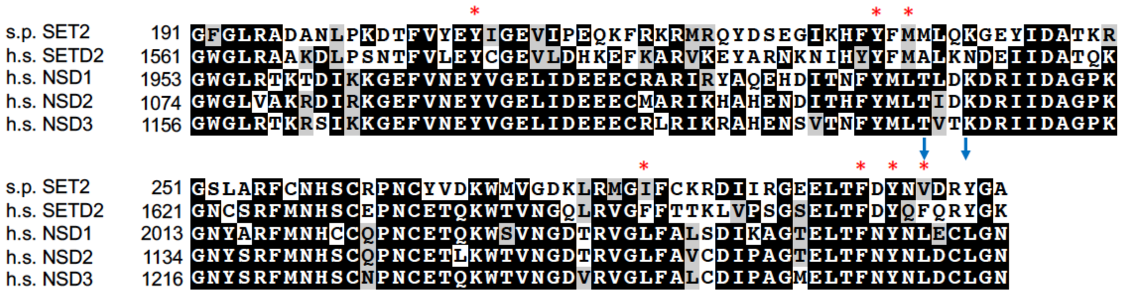

Extended data Figure 4. Sequence alignment of fission yeast (s.p.) and human (h.s.) H3K36 methyltransferases. Amino acids that mediates interaction with H3K36M are indicated by red asterisks, and amino acids that restrict the H3G34 access channel are indicated by blue arrows.

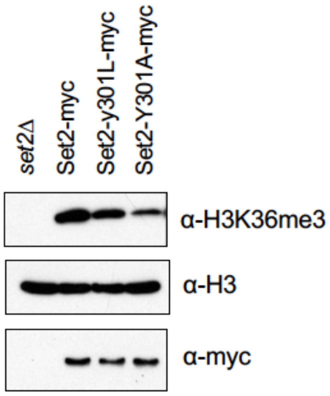

Extended data Figure 5. Western blot analyses of H3K36me3, H3, and Set2-myc levels.

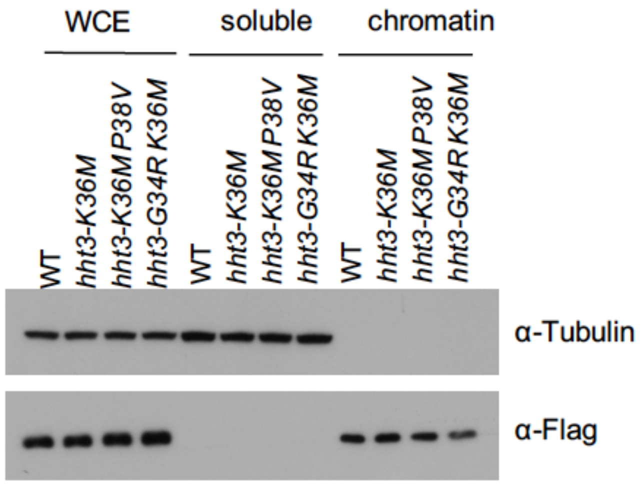

Extended data Figure 6. Chromatin fraction analyses to measure the incorporation of mutant histones into chromatin. Cell lysates (whole cell extract, WCE) were separated into soluble and chromatin-bound fractions. Western blot analyses were performed to measure Tubulin (cytoplasmic marker) or mutant histone levels in different fractions.

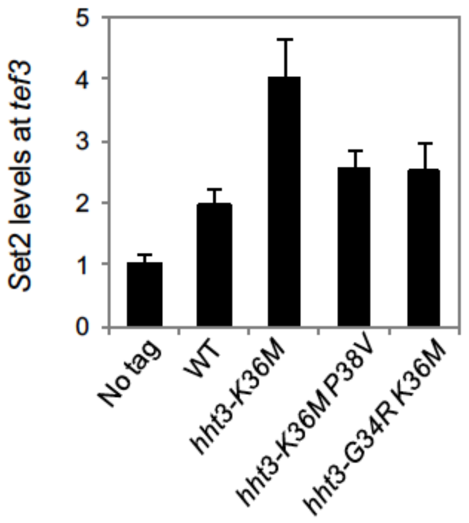

Extended data Figure 7. ChIP analyses of Set2-Flag levels at a target gene *tef3*, normalized to the silent mating-type locus. The mutant histone is GFP-tagged. Error bars represent the standard deviation of three replicates.

Extended data Table 1. Yeast strains used in this study

Name	*Mat*	*leu1*	*his2*	*ura4*	*ade6*	additional genotype
BR172	*mat1Msmt0*	*leu1*	*his2*	*DS/E*	*M210*	*otr::ura4*$^+$
SPJ4573	*mat1Msmt0*	*leu1*	*his2*	*DS/E*	*M210*	*hht3-Flag::hphMX6*
SPJ4729	*mat1Msmt0*	*leu1*	*his2*	*DS/E*	*M210*	*hht3-Flag::hphMX6 set2Δ::natMX6*
SPJ4647	*mat1Msmt0*	*leu1*	*his2*	*DS/E*	*M210*	*hht3-K36M-Flag::hphMX6*
SPJ4767	*mat1Msmt0*	*leu1*	*his2*	*DS/E*	*M210*	*hht3-K36R-Flag::hphMX6*
SPJ4768	*mat1Msmt0*	*leu1*	*his2*	*DS/E*	*M210*	*hht3-G34R-Flag::hphMX6*
SPJ4739	*mat1Msmt0*	*leu1*	*his2*	*DS/E*	*M210*	*hht3-G34V-Flag::hphMX6*
SPJ4813	*mat1Msmt0*	*leu1*	*his2*	*DS/E*	*M210*	*hht3-G34W-Flag::hphMX6*
SPJ4820	*mat1Msmt0*	*leu1*	*his2*	*DS/E*	*M210*	*hht3-G34L-Flag::hphMX6*
SPJ4771	*mat1Msmt0*	*leu1*	*his2*	*DS/E*	*M210*	*hht3-G34A-Flag::hphMX6*
SPJ4787	*mat1Msmt0*	*leu1*	*his2*	*DS/E*	*M210*	*hht3-G34C-Flag::hphMX6*
SPJ4783	*mat1Msmt0*	*leu1*	*his2*	*DS/E*	*M210*	*hht3-G34S-Flag::hphMX6*
SPJ4779	*mat1Msmt0*	*leu1*	*his2*	*DS/E*	*M210*	*hht3-G34D-Flag::hphMX6*
SPJ4775	*mat1Msmt0*	*leu1*	*his2*	*DS/E*	*M210*	*hht3-G34N-Flag::hphMX6*
SPJ4799	*mat1Msmt0*	*leu1*	*his2*	*DS/E*	*M210*	*hht3-G34T-Flag::hphMX6*
SPJ4735	*mat1Msmt0*	*leu1*	*his2*	*DS/E*	*M210*	*hht3-K36MP38V-Flag::hphMX6*
SPJ4751	*mat1Msmt0*	*leu1*	*his2*	*DS/E*	*M210*	*hht3-G34RK36M-Flag::hphMX6*
CS567	*mat1Msmt0*	*leu1*	*his2*	*DS/E*	*M210*	*set2-myc::kanMX6*
CS568	*mat1Msmt0*	*leu1*	*his2*	*DS/E*	*M210*	*hht3-Flag::hphMX6 set2-myc::kanMX6 otr::ura4*$^+$
CS556	*mat1Msmt0*	*leu1*	*his2*	*DS/E*	*M210*	*hht3-K36M-Flag::hphMX6 set2-myc::kanMX6 otr::ura4*$^+$
CS649	*mat1Msmt0*	*leu1*	*his2*	*DS/E*	*M210*	*hht3-K36MP38V-Flag::hphMX6 set2-myc::kanMX6 otr::ura4*$^+$
CS647	*mat1Msmt0*	*leu1*	*his2*	*DS/E*	*M210*	*hht3-G34RK36M-Flag::hphMX6 set2-myc::kanMX6 otr::ura4*$^+$
CS112	*mat1Msmt0*	*leu1*	*his2*	*DS/E*	*M210*	*hht3-GFP:kanMX6*
JW3464	*mat1Msmt0*	*leu1*	*his2*	*DS/E*	*M210*	*hht3-GFP::kanMX6 set2-Flag::kanMX6*
JW3411	*mat1Msmt0*	*leu1*	*his2*	*DS/E*	*M210*	*hht3-K36M-GFP::natMX6 set2-Flag::kanMX6 otr::ura4*$^+$
JW3415	*mat1Msmt0*	*leu1*	*his2*	*DS/E*	*M210*	*hht3-K36MP38V-GFP::natMX6 set2-Flag::kanMX6 otr::ura4*$^+$
JW3428	*mat1Msmt0*	*leu1*	*his2*	*DS/E*	*M210*	*hht3-G34RK36M-GFP::natMX6 set2-Flag::kanMX6 otr::ura4*$^+$

Extended data Table 2. DNA oligos used in this study.

primer name	sequence	annotation
cenH-RT-D	TTCAAGTCTTCTTTATACGTTTGC	for ChIP-qPCR of primer set at silent mating-type region
cenH-RT-C	CCATAGTAGTATGGCTATGAATG	
tef3-RT-R	TGCGACAGATTGCTTAACTG	for ChIP-qPCR of primer set at the *tef3* locus
tef3-RT-S	GTAAAACTAGTTGGTAAAAGGG	
